# Short Physical Performance Battery for cardiovascular disease inpatients: implications for critical factors and sarcopenia

**DOI:** 10.1038/s41598-017-17814-z

**Published:** 2017-12-12

**Authors:** Tomohiro Yasuda, Toshiaki Nakajima, Tatsuya Sawaguchi, Naohiro Nozawa, Tomoe Arakawa, Reiko Takahashi, Yuta Mizushima, Satoshi Katayanagi, Kazuhisa Matsumoto, Shigeru Toyoda, Teruo Inoue

**Affiliations:** 1 Department of Cardiovascular Medicine, School of Medicine, Dokkyo Medical University, Tochigi, Japan; 2 Heart Center, Dokkyo Medical University Hospital, Tochigi, Japan; 3 Department of Rehabilitation, Dokkyo Medical University, Tochigi, Japan; 4 School of Nursing, Seirei Christopher University, Shizuoka, Japan

## Abstract

We examined the relationship between Short Physical Performance Battery (SPPB) and clinical and laboratory factors and the effect of sarcopenia and sarcopenic obesity (SO) on clinical and laboratory factors for cardiovascular disease (CVD) inpatients. CVD male (*n* = 318) and female (*n* = 172) inpatients were recruited. A stepwise multiple-regression analysis was performed to predict total SPPB scores and assess clinical and laboratory factors (physical characteristics, functional and morphological assessments, etc.). Each test outcome were compared among sarcopenia, SO and non-sarcopenic groups. To predict total SPPB scores, the predicted handgrip, Controlling Nutritional Status score, % body fat, anterior mid-thigh muscle thickness, standing height and systolic blood pressure were calculated for males and anterior mid-thigh MTH, BMI, knee extension and fat mass were calculated for females. There were no differences in blood pressure, total SPPB scores and functional assessments between sarcopenia and SO groups for CVD male and female inpatients. In conclusion, the physical performance of CVD inpatients can be predicted by nutritional, functional, clinical and anthropometric variables, regardless the gender and the presence of sarcopenia. Furthermore, the presence of sarcopenia has a negative effect on the clinical and laboratory factors, but there is a difference in impact between sarcopenia and SO regardless the gender.

## Introduction

Cardiovascular disease (CVD) is a major contributor to the global burden of diseases^1,2^. In addition, CVD (heart failure and stroke, etc.) often causes disuse muscle atrophy in the acute and subacute phases, which increases the likelihood of wheelchair or bedridden state in the chronic phase^3–5^. Consequently, the progression of muscle atrophy in lower extremity function in CVD inpatients leads to a high need for medical and nursing care.

The Short Physical Performance Battery (SPPB), a brief performance battery based on a timed short distance walk, repeated chair stands, and a set of balance tests, is a validated assessment tool for measuring lower extremity function that is widely used in both clinical and research settings^6,7^. This means of assessment offers relative ease of use, perceived potential for implementation in clinical practice, and a good relationship with physical activity levels and walking disability in a variety of patients or older adults. In fact, a recent study demonstrated that the SPPB is an effective assessment tool for strength and lower extremity morphological evaluation for middle-aged and older cardiovascular disease patients (mixed inpatients and outpatients)^8^. It has also been found to predict mortality, hospitalization rate, and a variety of comorbid disease conditions^7^. However, a previous study of older inpatients reported that approximately 88% were malnourished and approximately 12% were at risk for malnutrition^9^. In a SPPB study for older inpatients, therefore, it is particularly necessary to carry out a comprehensive analysis (muscle strength, muscle morphology, nutrition status and body composition, etc.).

The assessment of body composition is commonly performed by quantifying fat mass and fat-free mass (e.g., skeletal muscle mass) components. These components are often utilized to assess the risk for adverse health outcomes in a variety of conditions^10^. In addition, some studies have demonstrated that sarcopenia, the age-related loss of skeletal muscle mass and strength^11^, and also obesity, an increase in fat mass^12^, increase the risk of mortality. Recently, sarcopenia is found to often co-occur with an increase in fat mass, termed sarcopenic obesity, which may carry the cumulative risk derived from each of the two individual body composition factors^13,14^. Accordingly, recent clinical studies have focused on health outcomes in sarcopenic obesity as well as sarcopenia^15,16^.

Thus, the purpose of this study was to examine the relationship between the SPPB and clinical and laboratory factors and the effect of sarcopenia and sarcopenic obesity on clinical and laboratory factors in CVD inpatients.

## Results

### The physical characteristics and clinical data

**Figure 1:**
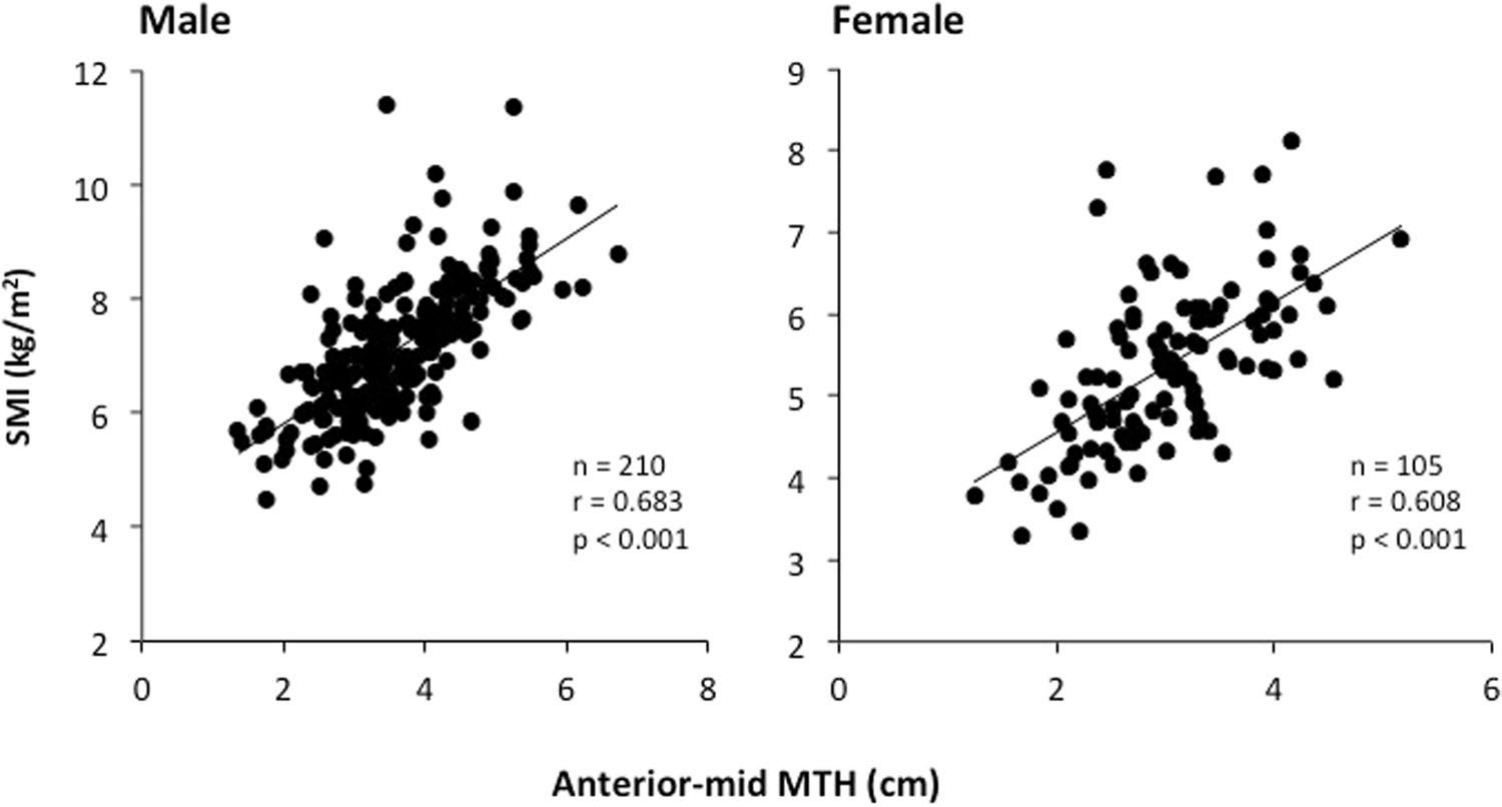
Relationships between anterior-mid muscle thickness (MTH) and skeletal muscle index (SMI) of CVD male and female inpatients.

**Figure 2:**
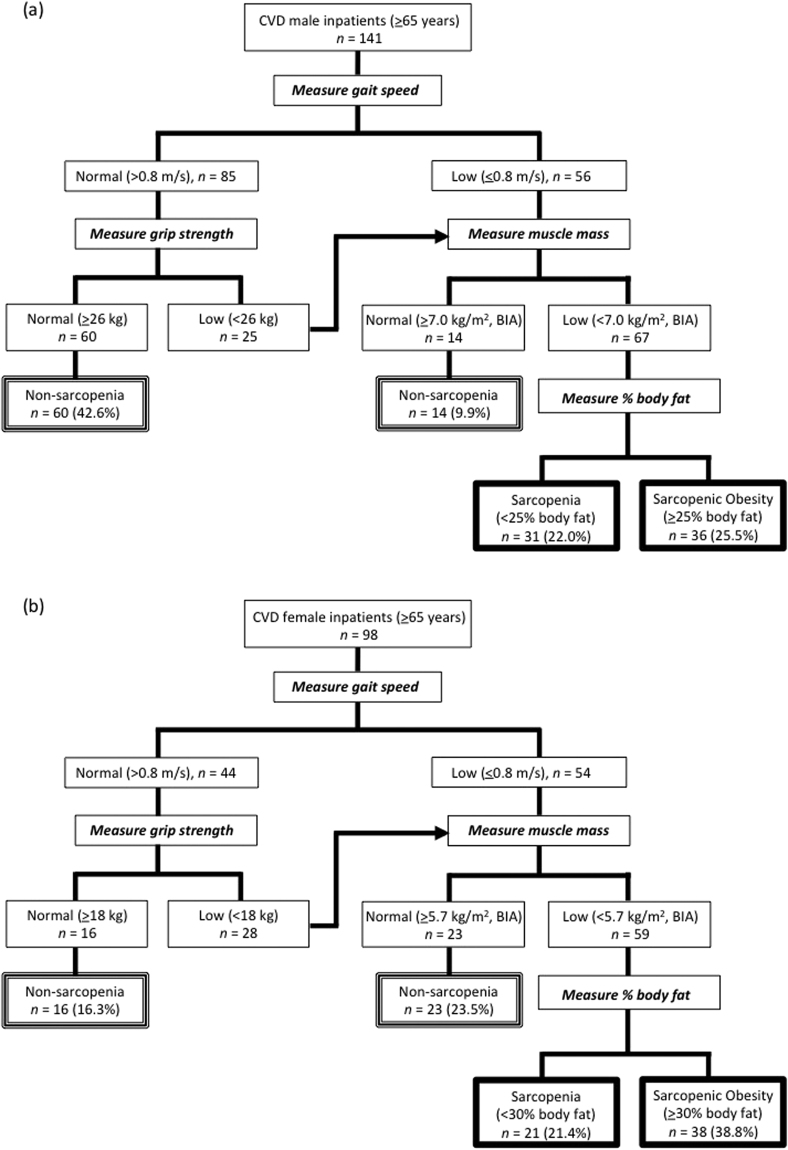
Diagnostic algorithm for Sarcopenia and Sarcopenic Obesity of CVD male (**a**) and female (**b**) inpatients.

Age and % body fat were greater (*P* < 0.01) in females than in males, but standing height, body weight, and BMI were greater (*p* < 0.01) in males than in females (Table 1). SPPB, functional and morphological assessments are shown in Table 2. Some subjects failed to perform the chair stand test (19 males and 17 females) and gait test (1 male and 2 females). In addition, BIA (InBody device) was contraindicated for 11 males and 15 females. Anterior-mid MTH correlated positively with SMI of males (r = 0.685, *p* < 0.0001) and females (r = 0.608, *P* < 0.0001) and the whole sample (r = 0.691, *p* < 0.0001) (Fig. 1). Total SPPB score, functional, and morphological assessments were greater (*p* < 0.01) in males than in females. Unlike the sarcopenia, the proportion of SO assessments for CVD patients was larger in females than in males (Fig. 2). In both CVD male and female inpatients, body weight, BMI, % body fat, and body fat mass were greater (*p* < 0.05) in SO than in sarcopenia. There were no differences (*p* > 0.05) in systolic and diastolic blood pressures, CRP, total SPPB scores and functional assessments between the sarcopenia and the SO groups for CVD male and female inpatients (Table 3).

**Table 1:** The physical characteristics and clinical data.

	Male (*n* = 318)	Range	Female (*n* = 172)	Range
Age, years	68.4 (13.0)	22–90	75.0 (10.8)**	40–96
Standing height, m	164.6 (6.5)**	147–190	149.0 (7.0)	127–173
Body weight, kg	64.0 (12.8)**	36.9–108.0	50.1 (10.7)	29.5–93.8
BMI, kg/m^2^	23.6 (3.9) **	13.8–36.5	22.3 (4.2)	14.1–37.9
% body fat	26.8 (7.7)	6.7–49.0	33.3 (10.1)**	8.0–53.6
Fat mass, kg	17.8 (7.6)	2.7–50.6	17.5 (8.0)	2.4–50.0
Systolic BP, mmHg	115 (16)	73–159	112 (16)	77–164
Diastolic BP, mmHg	65 (11)	40–96	64 (11)	35–93
CONUT score, unit	3.6 (2.8)	0–12	3.7 (2.5)	0–10
BNP, pg/mL	536 (703)	3–3843	553 (812)	7–5015
Specific diseases, *n*				
Cardiovascular surgery patients, *n*				
CABG and/or non-CABG	86	—	37	—
Aortic dissection and aneurysm	42	—	21	—
ASO	9	—	3	—
Others (VAD, ASD, *et al*.)	3	—	4	—
TAVI	1	—	11	—
Patients with internal cardiovascular diseases				
CHF	95	—	65	—
CHD (AMI, ACS, PCI *et al*.)	79	—	27	—
Others (PE etc.)	3	—	4	—

Data are given as mean (standard deviation). BMI, Body mass index. BNP, brain natriuretic peptide. BP, blood pressure. CONUT, Controlling Nutrition Status. SMI, skeletal muscle mass index. CABG, coronary artery bypass grafting. Non-CABG, heart valve replacement and repair. VAD, ventricular assist device. VSD, ventricular septal defect. ASO, ateriosclerosis obliterans. TAVI, tanscatheter aortic valve implantation. CHF, congestive heart failure. CHD, coronary heart disease. AMI, acute myocardial infarction. ACS, acute coronary syndrome. PCI, percutaneous coronary intervention. PE, pulmonary embolism. **p < 0.01, Male vs. Female.

**Table 2:** Short physical performance battery (SPPB), functional, and morphological assessments.

	Male(n =318)	Range	Female (*n* = 172)	Range
SPPB score				
Balance test	3.6 (0.8)**	0–4	3.1 (1.2)	0–4
Gait test	3.4 (0.9)**	0–4	3.0 (1.0)	1–4
Chair stand test	2.7 (1.4)**	0–4	2.0 (1.4)	0–4
Total SPPB	9.7 (2.7)**	0–12	8.2 (3.0)	1–12
Functional assessment				
Handgrip, kg	28.3 (8.5)**	10.4–52.5	15.2 (4.6)	0.0–28.3
Knee extension, kg	25.6 (12.1)**	5.2–60.2	13.7 (6.7)	2.0–45.4
Morphological assessment				
Fat-free mass, kg	46.2 (7.9)**	17.6–71.1	32.5 (5.3)	17.8–53.9
Skeletal muscle mass, kg	24.9 (4.7)**	15.3–39.1	16.8 (3.5)	11.1–37.0
SMI, kg/m^2^	7.08 (1.17)**	4.48–11.40	5.30 (1.00)	3.25–8.11
Mid-thigh girth, cm	45.4 (5.9)**	30.1–61.0	42.3 (6.2)	29.0–62.4
Anterior mid-thigh MTH, cm	3.62 (0.98)**	1.36–6.72	3.00 (0.73)	1.24–5.17
Posterior mid-thigh MTH, cm	5.58 (1.07)**	2.58–8.62	4.91 (0.99)	2.02–8.75

Data are given as mean (standard deviation). MTH, muscle thickness. SMI, skeletal muscle mass index. **p < 0.01, Male vs. Female.

**Table 3:** Comparison among Sarcopenia, Sarcopenic Obesity (SO) and non-sarcopenic (NS) groups in cardiovascular disease male and female inpatients (≥65 years).

	Male (*n* = 141)			Female (*n* = 98)		
	Sarcopenia	SO	NS	Sarcopenia	SO	NS
	Mean (SD)	Mean (SD)	Mean (SD)	Mean (SD)	Mean (SD)	Mean (SD)
	*n* = 31	*n* = 36	*n* = 74	*n* = 21	*n* = 38	*n* = 39
Age, years	77.2 (6.5)^##^	79.0 (5.8)^¶¶^	72.5 (6.0)	79.9 (8.0) ^#^	79.3 (6.2)^¶¶^	74.7 (6.4)
Systolic BP, mmHg	116 (16)	115 (14)	116 (18)	110 (18)	110 (16)^¶^	120 (15)
Diastolic BP, mmHg	62 (13)	62 (12)	65 (10)	61 (11)	64 (9)	67 (10)
CRP, mg/dl	4.5 (6.3)^*,##^	2.8 (5.8)	1.6 (2.5)	1.8 (3.0)	1.5 (2.9)	1.3 (3.7)
BNP, pg/mL	802 (778)^##^	498 (644)^¶^	362 (510)	690 (537)^##^	504 (563)^¶^	269 (555)
CONUT score, unit	5.9 (2.7)^*,##^	4.0 (2.9)	2.9 (2.6)	3.8 (2.6)	3.5 (2.2)	2.8 (2.5)
SPPB score						
Balance test	3.5 (0.7)	3.3 (1.1)^¶^	3.7 (0.8)	3.1 (0.9)^##^	2.8 (1.0)^¶¶^	3.4 (0.8)
Gait test	2.9 (1.0)^##^	2.8 (0.9)^¶¶^	3.8 (0.6)	2.8 (1.0) ^#^	2.8 (1.1)^¶^	3.4 (0.8)
Chair stand test	1.7 (1.3)^##^	1.9 (1.5)^¶¶^	3.3 (0.9)	1.4 (1.2)^##^	1.8 (1.3)^¶^	2.5 (1.4)
Total SPPB	8.2 (2.1)^##^	7.9 (2.5)^¶¶^	10.8 (1.9)	7.4 (2.4)^##^	7.6 (3.1)^¶^	9.4 (2.6)
Functional assessment						
Handgrip, kg	20.9 (4.6)^##^	20.7 (4.7)^¶¶^	31.2 (6.4)	12.7 (4.2)^##^	13.3 (3.2)^¶¶^	18.3 (3.8)
Knee extension, kg	17.6 (6.5)^##^	16.4 (6.6)^¶¶^	28.7 (11.5)	8.7 (3.5)^**,##^	12.3 (5.4)^¶¶^	17.7 (5.9)
Morphological assessment						
Height, m	1.61 (0.05)^¶¶^	1.60 (0.06)^¶¶^	1.64 (0.05)	1.48 (0.07)	1.46 (0.07)^¶¶^	1.50 (0.05)
Body weight, kg	53.0 (7.0)^##,**^	60.0 (5.8)^¶¶^	65.8 (9.4)	40.9 (7.6)^**,##^	48.9 (6.6)^¶¶^	55.3 (8.8)
BMI, kg/m^2^	20.2 (2.5)^##,**^	23.2 (2.3)	24.1 (3.2)	17.9 (2.8)^**,##^	22.8 (3.3)^¶^	24.1 (3.5)
% body fat	19.7 (3.7)^##,**^	32.8 (4.4)^¶¶^	27.4 (6.1)	20.3 (6.7)^**,##^	39.3 (6.5)^¶^	35.5 (7.9)
Fat-free mass, kg	40.6 (3.9)^*,##^	38.2 (4.9)^¶¶^	47.3 (5.9)	30.2 (4.8)^##^	28.9 (3.3)^¶¶^	35.0 (3.6)
Body fat mass, kg	10.3 (2.6)^##,**^	19.1 (3.7)	18.2 (5.6)	9.5 (6.0)^##,**^	19.2 (5.3)	20.0 (7.0)
Skeletal MM, kg	21.2 (2.4)^##^	20.4 (2.0)^¶¶^	25.6 (3.4)	15.3 (2.2)^##^	14.6 (1.9)^¶¶^	18.1 (2.0)
SMI, kg/m^2^	6.02 (0.67)^##^	6.09 (0.53)^¶¶^	7.50 (1.05)	4.62 (0.60)^##^	4.72 (0.67)^¶¶^	6.09 (0.73)
Mid-thigh girth, cm	39.2 (3.0)^**,##^	42.3 (3.5)^¶¶^	46.0 (4.0)	36.9 (6.2)^*,##^	41.5 (3.6)^¶¶^	46.1 (5.0)
Anterior MTH, cm	2.67 (0.54)^*,##^	2.97 (0.67)^¶¶^	3.74 (0.71)	2.46 (0.52)^##^	2.80 (0.70)^¶¶^	3.35 (0.58)
Posterior MTH, cm	4.91 (0.94)^##^	5.10 (1.04)^¶¶^	5.80 (0.89)	4.63 (0.73)^##^	4.78 (1.26)^¶¶^	5.27 (0.78)

Data are given as mean (standard deviation). BNP, brain natriuretic peptide. BP, blood pressure. CONUT, Controlling Nutrition Status. CRP, C-reactive protein. MM, muscle mass, MTH, mid-thigh muscle thickness. SMI, skeletal muscle mass index. ^**^p < 0.01, ^*^p < 0.05, Sarcopenia vs. SO groups. ^##^p < 0.01, ^#^p < 0.05^,^ Sarcopenia vs. Non-sarcopenic groups. ^¶¶^p < 0.01, ^¶^p < 0.05, SO vs. Non-sarcopenic groups.

### The relationship between SPPB and clinical and laboratory factors

To predict total SPPB scores, the predicted handgrip, CONUT, % body fat, anterior mid-thigh MTH, standing height and diastolic BP were calculated for males (total SPPB scores = 0.101 × handgrip − 0.199 × CONUT − 0.110 × % body fat + 0.782 × anterior mid-thigh MTH −0.072 × standing height + 0.029 × diastolic BP + 17.836) (*n* = 318, R^2^ = 0.523, *p* < 0.05) and anterior mid-thigh MTH, BMI, knee extension and fat mass were calculated for females (total SPPB scores = 1.902 × anterior mid-thigh MTH −0.627 × BMI + 0.157 × knee extension + 0.223 × fat mass + 10.940) (*n* = 172, R^2^ = 0.411, *p* < 0.05).

## Discussion

The main findings of this study were as follows: First, anterior mid-thigh MTH can predict total SPPB scores for both CVD male and female inpatients. Second, non-sarcopenic group was superior for morphological and functional assessments compared with sarcopenia and SO groups, and there were slightly differences between the two groups in assessment for CVD male and female inpatients.

A previous study reported that impaired mobility was reflected by total SPPB scores of less than 10 and those with total SPPB scores of 7–9 were 1.6 to 1.8 times more likely to become disabled^17,18^. In addition, total SPPB scores of 4–6 were 4.2 to 4.9 times more likely to have disability in the activities of daily living or mobility-related disability at 4 years. Therefore, the SPPB assessment was established as a disability evaluation for older adults, healthy elderly and for some disability conditions^6,19,20^. In this study, the average score (9.7 for males and 8.2 for females) and the distribution (0–3: 6% and 5%, 4–6: 20% and 20%, 7–9: 31% and 25%, 10–12: 43% and 49% for male and female inpatients, respectively) of total SPPB scores were almost the same as the previous CVD study (score 9.0; distribution 0–3: 0%, 4–6: 17%, 7–9: 25%, 10–12: 50% for mixed male and female inpatients)^8^. Although a previous study investigated whether SPPB can be validated as an assessment tool for muscle strength and morphology in various physical condition (12 inpatients, 12 outpatients and 12 healthy subjects), a stepwise multiple-regression analysis could be applied to the predictor anterior mid-thigh muscle thickness to predict total SPPB scores for CVD in both studies. Taken together, these results suggested that the total SPPB scores could be evaluated by morphological assessment in knee extensor muscles for middle-aged and older adults regardless of male/female and CVD outpatient/inpatient.

In general, SMI assessed by BIA and DXA is used extensively in sarcopenia criteria as in European Working Group on Sarcopenia in Older People (EWGSOP)^21^ and AWGS^22^. However, we could not measure the SMI for 26 inpatients (11 males and 15 females), because individuals with cardiac pacemakers are generally regarded as a contraindication to BIA, DXA and MRI measurements. In contrast, MTH assessed by ultrasound is also used as a muscle mass in clinical medicine and sports science, and there is no contraindication even for individuals with cardiac pacemakers. In addition, there was a high correlation coefficient (r = 0.685, *p* < 0.0001 for males and r = 0.608, *p* < 0.0001 for females) between anterior mid-thigh MTH (mainly dependent on quadriceps muscles) and SMI in this study (Fig. 1). Furthermore, previous studies revealed that sarcopenia is muscle specific and greater quadriceps muscle loss was found in older adults^23,24^. These results indicated that additional research into anterior mid-thigh MTH assessed by ultrasound on sarcopenia criteria is worthy of attention.

There was little relationship between the total SPPB scores and CONUT score in this study. The CONUT score was 3.6 for male and 3.7 for female inpatients and the prevalence of malnutrition (mild [2–4]: 40% and 34%; moderate [5–8]: 26% and 37%; severe [9–12]: 7% and 4% for male and female, respectively) was 73–75%. These results were similar to those in a previously reported studies in inpatients with acute or chronic heart failure (78% for AHF and 60–69% for CHF)^25,26^. This appears that prognostic value of malnutrition assessed by CONUT score may not be a decisive factor in the assessment of SPPB score for CVD male inpatients.

A previous study reported that SO is associated with all-cause mortality and also with greater CVD mortality, largely because of their association with blood pressure, blood lipids and inflammation^27^. In this study, there was no difference in blood pressures, inflammation, and functional assessment between the sarcopenia and the SO groups for CVD male and female inpatients. Additionally, the CONUT score and the knee extensor muscle morphology for the SO group were either equal to or surpassed those of the sarcopenia group. Therefore, it is highly likely that the SO group induced greater cardiovascular mortality and all-cause mortality was independent of functional assessment, as well as blood pressure and inflammation, although the mortality for the sarcopenia and the SO groups was not investigated. Additional research into these issues is needed.

The present study has some limitations. First, it was very difficult to evaluate the various assessments within the same hospital admission for the inpatients. Second, SPPB assessment could not be evaluated for severely impaired mobility patients because some subjects failed to perform the chair stand test (19 males and 17 females) and the gait test (1 male and 2 females). Third, some data were missing due to impossible measurement (method discussed above), sudden discharge from hospital, acute deterioration, contraindication and others for the inpatients. Additional research into these issues is needed.

In conclusion, the physical performance of CVD inpatients can be predicted by nutritional, functional, clinical and anthropometric variables, regardless the gender and the presence of sarcopenia. Furthermore, the presence of sarcopenia has a negative effect on the clinical and laboratory factors, but there is a difference in impact between sarcopenia and SO regardless the gender.

## Methods

### Participants

Four hundred-ninety (aged 22 to 96 years) male (*n* = 318) and female (*n* = 172) inpatients with cardiovascular disease volunteered to participate in the study and were selected according to the exclusion criteria (i.e. cerebrovascular disease patients and those undergoing arthroscopic joint surgery) (Table 1). In addition, volunteers who suffered from a chronic disease such as severe orthopedic disorders, or cognitive dysfunction were excluded from the study. All participants had undergone complete chemistry and hematologic evaluation and were informed of the risks associated with involvement in the study and signed an informed consent document before participation. All participants were recruited through oral communications in the Dokkyo Medical University Hospital. The principles of the World Medical Association Declaration of Helsinki and the American College of Sports Medicine Guidelines for Use of Human Subjects were adopted in this study. The study was approved by the Ethics Committee of the Dokkyo Medical University, and informed assent consent were obtained from the participants. All data were analyzed by the same author (a specialist in exercise physiology research for more than 15 years), who was blinded to all of the measurements.

### Short physical performance battery

Participants performed the Short Physical Performance Battery (SPPB) according to the National Institute on Aging protocol. The tests were performed in the following sequence: a) standing balance tests, b) gait test (4 m), and c) chair stand test (5 repetitions). The standing balance portion requires participants to maintain, for 10 seconds each, stances with their feet placed side by side, semi-tandem, and in tandem. The scores ranged from 0 to 4 (maximum performance). The gait test measured the time needed to walk 4 m at a typical pace. The chair stand required participants to rise from a steel chair (0.40 m height and 0.30 m depth) with their arms across their chest, five times. Categorical scores (range: 0 to 4) for both the gait and the chair stand tests were based on timed quartiles established previously in a large population. Individuals who were unable to complete either the 4 m gait task or the 5 repetitions chair stand test received a score of 0. The sum of the three components comprised the final SPPB score, with a possible range from 0 to 12. A score of 12 indicated the highest degree of lower extremity function^6,28^.

### Maximum voluntary isometric contraction

Maximum voluntary isometric contraction (MVIC) of the handgrip was determined using a factory-calibrated hand dynamometer (TKK 5401, TAKEI Scientific Instruments Co., Ltd., Tokyo, Japan). All of the subjects were instructed to maintain an upright standing position, arms at their side, holding the dynamometer in the right hand with the arm at a right angle and the elbow held at the side of the body. The size of the dynamometer handle was set so that it felt comfortable to the subject while squeezing the grip. Each subject underwent 2 trials, and the best value of 2 trials was used for analysis.

MVIC of the knee extensors was determined using a digital handheld dynamometer (μTas MT-1, ANIMA Co., Ltd., Tokyo, Japan)^29^. The dynamometer pad used was 55 × 55 mm, and its front side was curved to fit the shape of the area of the extremity to be measured. Subjects were seated in a hard chair with their knees flexed 90° and their arms on their thighs. The dynamometer was placed perpendicular to the leg just above the malleoli. During all tests, the dynamometer was kept stable by the examiner using both hands and the subject’s leg was fixed by a belt to keep the knee flexed at 90°. Subjects were told to push against the dynamometer by attempting to straighten their leg. They were asked to build force gradually to a maximum voluntary effort. Each subject performed 2 trials with an interval of at least 2 min between the trials. The highest score was adopted for the individual data.

### Skeletal muscle index

The multi-frequency bioelectrical impedance analyzer (BIA), InBody S10 Biospace device (Biospacte Co., Ltd., Korea/Model JMW140) was used according to the manufacturer’s guidelines. BIA estimates body composition using the difference of conductivity of the various tissues based on the differences in their biological characteristics. Conductivity is proportional to water content, and more specifically to electrolytes, and it decreases as the cell approaches a perfect spherical shape. Adipose tissue is composed of round shaped cells and contains relatively little water compared to other tissues like muscle; therefore, conductivity isdecreased as body fat increases. In practice, electrodes are placed at 8 precise tactile-points of the body to achieve a multi-segmental frequency analysis. A total of 30 impedance measurements were obtained using 6 different frequencies (1, 5, 50, 250, 500, and 1000 kHz) at the 5 following segments of the body: right and left arms, trunk, right and left legs^30^. The measurements were carried out while the subjects rested quietly in the supine position, with their elbows extended and relaxed along their trunk. Percent body fat, fat-free mass and skeletal muscle mass were recorded. Also, skeletal muscle index (SMI; AMM/height^2^, kg/m^2^) was measured as the sum of lean soft tissue of the two upper limbs and two lower limbs (appendicular muscle mass: AMM).

### Muscle thickness

After thigh length measurements using anatomic landmarks, all measurement sites were marked with a marker pen and then mid-thigh (at 50% between the lateral condyle of the femur and the greater trochanter) girth and mid-thigh muscle thickness were measured using a tape measure on the right side of the body^31^. Ultrasound evaluation of muscle thickness (MTH) was performed by using a real-time linear electronic scanner with a 10.0-MHz scanning head and Ultrasound Probe (L4–12t-RS Probe, GE Healthcare Japan) by using LOGIQ e ultrasound (GE Healthcare Japan). The scanning head was coated with a water-soluble transmission gel to provide acoustic contact without depressing the dermal surface. The subcutaneous adipose tissue-muscle interface and the muscle-bone interface were identified from the ultrasonic image. The perpendicular distance from the adipose tissue-muscle interface to the muscle-bone interface was considered to represent MTH. Briefly, the measurements were carried out while the subjects stood with their elbows extended and relaxed^31^.

### Controlling nutritional status score

The Controlling Nutritional Status (CONUT) score, which is calculated by the serum albumin concentration (range: 0 to 6), the total peripheral lymphocyte count (range: 0 to 3), and the total cholesterol concentration (range: 0 to 3), was developed as a screening tool for early detection of poor nutritional status. The sum of the three components comprised the final CONUT score, with a possible range from 0 to 12. A score of 12 indicated the poorest nutritional status^32^. Use of the CONUT score has advantages, such as simplicity and cost effectiveness^32,33^.

### Definition of sarcopenia and sarcopenic obesity

In this study, sarcopenia was defined according to the Asian Working Group for Sarcopenia (AWGS)^22^ criteria (age, ≥65 years; handgrip, <26 kg for males and <18 kg for females; gait speed, ≤0.8 m/sec; SMI, <7.0 kg/m^2^ for males and <5.7 kg/m^2^ for females). Sarcopenia obesity (SO) was considered to be the combination of sarcopenia and obesity (% body fat >25% for males and >30% for females)^34–37^.

### Statistical analyses

Results are expressed as mean ± standard deviation for all variables. All data were analyzed using JMP v.12.0 for Mac (SAS Institute Inc., Tokyo, Japan). Pearson product correlations of total SPPB scores and variable factors were also statistically quantified. When the data were not normally distributed, non-parametric statistical analysis (Wilcoxon signed rank test) was used to identify differences in sarcopenia, SO and non-sarcopenic groups. Since the value of events per variable = 10 seems most prudent for regression analysis^38^, 17 variable factors were acceptably for both genders (*n* = 318 and 172 for male and female inpatients, respectively). In addition, the variance inflation factor (VIF) were used to determine the degree of multi-collinearity of the *i-*th independent variable with other independent variables for all hierarchal regression models^39^. Multi-collinearity between variables was defined as a VIF ≥ 10, and fat free-mass was excepted for male in this study. Based on the result of VIF, a stepwise multiple-regression analysis (method of increasing and decreasing the variables, criterion was set at *p* < 0.05) was performed to predict SPPB scores and variable factors (age, standing height, body weight, BMI, % body fat, systolic and diastolic blood pressures, resting heart rate, handgrip, knee extension, fat free-mass (used exclusively for female), skeletal muscle mass, SMI, mid-thigh girth, anterior and posterior mid-thigh muscle thickness and CONUT score). Consequently, the predicted variables, coefficients and intercept coefficients were automatically picked out by the JMP software. Statistical significance was set at *p* < 0.05.
